# Interaction-Limited Aggregation: Fine-Tuning the Size of pNIPAM Particles by Association with Hydrophobic Ions

**DOI:** 10.1021/acs.macromol.3c00132

**Published:** 2023-03-17

**Authors:** Jordi Faraudo, Arturo Moncho-Jordá, Delfi Bastos-González, Carlos Drummond

**Affiliations:** † Institut de Ciència de Materials de Barcelona (ICMAB-CSIC), Campus de la UAB, E-08193 Bellaterra, Spain; ‡ Biocolloid and Fluid Physics Group, Department of Applied Physics, University of Granada, Avenida Fuentenueva 2, E-18071 Granada, Spain; ¶ Institute Carlos I for Theoretical and Computational Physics, Facultad de Ciencias, Universidad de Granada, Campus Fuentenueva S/N, 18071 Granada, Spain; § Centre de Recherche Paul Pascal Université Bordeaux, CNRS, CRPP, UMR 5031, F-33600 Pessac, France

## Abstract

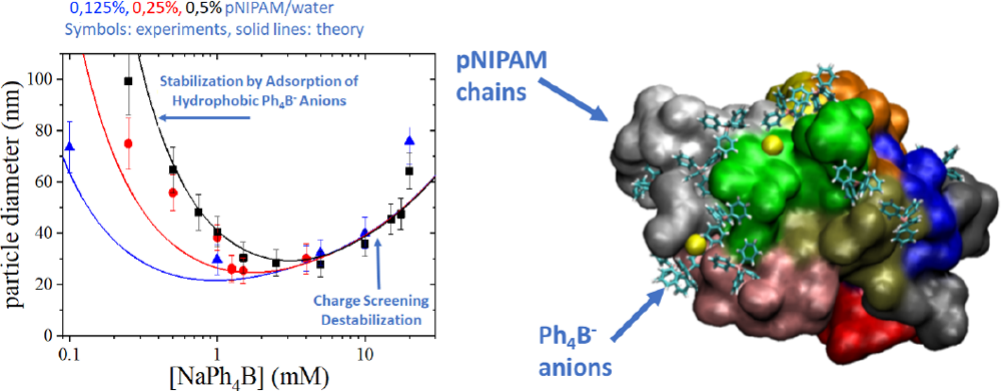

We have investigated the formation of stable clusters of poly(*N*-isopropylacrylamide) (pNIPAM) chains in water at temperatures above the lower critical solution temperature (LCST), induced by the presence of sodium tetraphenylborate, NaPh_4_B. The hydrophobic Ph_4_B^–^ ions interact strongly with the pNIPAM chains, providing them with a net effective negative charge, which leads to the stabilization of pNIPAM clusters for temperatures above the LCST, with a mean cluster size that depends non-monotonically on salt concentration. Combining experiments with physical modeling at the mesoscopic level and atomistic molecular dynamic simulations, we show that this effect is caused by the interplay between the hydrophobic attraction between pNIPAM chains and the electrostatic repulsion induced by the associated Ph_4_B^–^ ions. These results provide insight on the significance of weak associative anion–polymer interaction driven by hydrophobic interaction and how this anionic binding can prevent macroscopic phase separation. Harvesting the competition between attractive hydrophobic and repulsive electrostatic interaction opens avenues for the dynamic control of the formation of well-calibrated polymer microparticles.

## Introduction

Phase separation in macromolecular solutions has been widely investigated. The process typically involves nucleation and growth of liquid polymer-rich droplets, followed by coalescence and droplet ripening, leading to macroscopic separation in a polymer-rich and a polymer-poor phase. The equilibrium state of the system depends on thermodynamic variables, but the evolution of the morphology of the system to this state may be limited by diffusion or hydrodynamic forces.^1^ Macromolecules sensitive to environmental conditions (e.g., temperature, pH, or ionic strength) constitute model systems to prompt and study controlled phase transitions, with multiple potential applications (e.g., drug delivery or material manufacturing). An archetypical example is the thermosensitive polymer poly(*N*-isopropylacrylamide), pNIPAM, which is water-soluble at low temperatures, but becomes insoluble at temperatures above the lower critical solution temperature (LCST).^2^ This reversible, entropy-driven (exothermic) transition is governed by the delicate balance between weak water–polymer and intra/interpolymer interactions.

In some cases, the loss of stability of macromolecular solutions does not carry on up to macroscopic separation; instead, polymer-rich drops of limited size are formed. Thus, the formation of long-lasting single-chain globules or multimolecular aggregates (mesoglobules) has been reported.^3−8^ Thermodynamic instability is clearly the driving force for rapid macromolecular aggregation and drop formation. However, it is not always apparent which factors hinder complete macroscopic phase separation, a process governed by the extension of intermolecular interactions. In the case of dilute polymer solutions (<0.1% w/w), it has been suggested that the small probability of interdrop collision is responsible for the stability of the mesoglobules.^6,8,9^ Indeed, it is always observed that larger polymer concentrations readily trigger macroscopic phase separation. Also, there are other possible stabilizing mechanisms such as electrostatic interactions due to the presence of charged terminal groups in the polymer chains (see ref (9) for a review).

In practice, it may be difficult to distinguish between kinetically frozen, long-lived, metastable aggregates and particles at thermodynamic equilibrium. However, the limited coalescence of macromolecular-rich droplets can be of interest to many processes. It can be used to enhance reaction rates by increasing the concentration of reactants (microreactors)^10^ and sequestration/isolation of harmful species. Similar schemes can be used for controlled release^11^ or water remediation.^12^ This process has also been associated with the origin of life.^13^ In this direction, it has been increasingly recognized that restricted liquid–liquid phase separation determines the formation and stability of a number of membraneless structures (organelles) governing essential cellular functions.^14,15^ For instance, controlled segregation of RNA and proteins, governed by specific and weak interactions, is necessary for the formation of nucleoli (for ribosome assembly), the formation of intranuclear compartments, or the appearance of stress granules in the cytoplasmic matrix.^16^ In many biological processes, it is crucial to understand how these organelles are stabilized against coalescence.

Despite its importance, the understanding of the mechanisms governing limited macromolecular association in phase separation is still incomplete. Most systems studied have been a multicomponent mixture of macromolecules where the association is driven by attractive electrostatic or specific interactions (often called complex coacervation), typically leading to the formation of micron-size liquid droplets.^17,18^ On the contrary, fewer studies on single-component separation of submicrometric drops have been reported. Simple coacervation refers to the limited separation of a single polymeric species, commonly in the presence of a triggering molecule. For instance, gelatin coacervates in the presence of alcohols because of decreasing solvent quality,^19^ and DNA can form dense aggregates by addition of spermine or cobalt hexamine, which screens the intermolecular electrostatic repulsive interaction.^20^ As another example of this single-component scenario, we have recently described how the phase separation of pNIPAM solutions can be dynamically controlled by adjusting the physicochemical environment. In particular, we reported that the presence of hydrophobic/associative ions hinders the phase separation of the system at temperatures above the LCST.^21^ The most interesting case corresponds to the addition of tetraphenyl borate anions (Ph_4_B^–^), which are known to have a marked chaotropic behavior,^22,23^ interacting strongly with any hydrophobic material^23,24^ including pNIPAM chains above the LCST.^25^ In these conditions, macromolecule-rich droplets of relatively uniform size are formed and stabilized at high temperatures and reversibly disassemble at low temperatures.^21^ We have shown that the stability of the liquid polymer-rich droplets is governed by electrostatic effects; as a consequence, it is progressively limited by increasing the concentration of nonassociating salts. Using this method, the limited coalescence and formation of calibrated polymer drops may open pathways for fine-tuning of particle size and reversible sequestration/compartmentalization. The dynamics of the process and the final particle size are strongly dependent on the type of ions present, and the salt/polymer concentration ratio.

The objective of this work is to elucidate the mechanisms underlying this limited drop coalescence. To this end we analyze further experimental data with a combination of mesoscopic physical modeling and molecular dynamic simulations, outlining a rational method for the dynamic control of formation and disappearance of size-calibrated pNIPAM-rich droplets. The article is organized as follows. We start considering new experimental studies on incomplete pNIPAM phase separation at temperatures above the LCST in the presence of different concentrations of the sodium salt of tetraphenylborate, Ph_4_B^–^, and for different pNIPAM concentrations, extending the work developed in ref (21). In addition, we carried out ion-specific electrode measurements to evaluate the interaction between pNIPAM and tetraphenyl anions at temperatures below the LCST. Then, we performed atomistic molecular dynamic (MD) simulations, which provided a detailed description of the interaction between pNIPAM chains and Ph_4_B^–^ in water. The results obtained in the MD simulations were employed in an analytical theoretical model, formulated at a coarse-grained level of description, that includes the main aspects of the physical process behind the limited pNIPAM aggregation. This model combines the destabilizing hydrophobic interaction between the polymer chains (at temperatures above the LCST) with the stabilizing electrostatic repulsion due to the associated anions. The model accurately predicts the major trends observed experimentally and the measured size of the pNIPAM-rich droplets, starting from the information gathered from MD simulations.

## Materials and Methods

### Materials

Sodium tetraphenylborate was obtained from Sigma-Aldrich. Poly(*N*-isopropylacrylamide) (ref 535311) was purchased from Sigma-Aldrich. Similar results were obtained with a second sample from Sigma-Aldrich (*M*_w_ = 10 000–15 000 g/mol, lot MKB68446V). All the products were of analytical grade and were used as received. Water used in all experiments was double distilled and deionized (DDI) with a Milli-Q water purification system (Millipore). pNIPAM/salt solutions were prepared at room temperature. For aging and particle size measurements, 2.5 mL of polymer solution was placed in borosilicate glass tubes (100 mm length, 1 mm wall thickness; VWR) and stored at 25 °C before measurement. pNIPAM aggregation was triggered by placing the tubes in a large-size bath at 45 °C. Samples were thermally equilibrated 15 min before starting the DLS measurements.

### Methods

#### Dynamic Light Scattering

Dynamic light scattering (DLS) measurements were performed using a BI-200SM motorized goniometer (Brookhaven Instruments) and a BI-9000AT digital autocorrelator (laser wavelength 633 nm; scattering angles between 30° and 145°). Particle sizes were determined from the measured intensity autocorrelation function of the scattering intensity using the method of cumulants. Similar results were obtained using a second DLS setup consisting of an ALV goniometer and an ALV-5000 digital correlator (laser wavelength 633 nm; scattering angle 30° to 145°).

#### Sodium-Ion-Selective Electrode

Measurements of sodium ion activity were carried out on a Consort C533 digital pH/mV meter and a sodium-ion-selective electrode (ISE) (Thermo Scientific combination electrode model 8611BNWP). Potential difference measurements were performed at a constant temperature (25 °C). The potential difference electromotive force (EMF) was recorded 2–3 min after electrode immersion, after a stationary value was achieved. Special care was taken to control the purity of the solutions investigated, in particular at low salt concentrations. All glassware was abundantly rinsed with water and ethanol and dried with filtered nitrogen gas before use. The ISE electrode was abundantly rinsed with distilled water before immersion in the solutions investigated, to avoid cross-contamination. NaPh_4_B concentration was varied between 0.5 and 10 mM. pNIPAM concentration was varied between 0 and 0.5% w/w. By using a sodium ISE, the measured potential is dominated by the activity of the sodium ions in solutions, *a*_Na_. If the interface is perfectly selective toward this ion and Nernst’s equation is followed, the measured EMF will be given by *E* = *E*_0_ + *S* log_10_(*a*_Na_). The slope, *S*, corresponds to *RT*/(*zF*) (59.18 mV for monovalent ions at 298 K), where *R* is the gas constant, *F* is the Faraday constant, and *z* is the valence of the ion.^26^ Thus, the measured EMF depends on the logarithm of the ionic activity. The measured EMF value is the addition of the different boundary potentials in the cell. Thus, the validity of the mentioned equation requires the system to be in thermodynamic equilibrium and that the value of the different contributions (other than the one determined by the activity of the sodium ions) remains constant. At low salt concentrations, a value of 1 can be assumed for the activity coefficient (defined as customary as the ratio between the ionic activity and the ionic concentration), and the EMF will be proportional to the logarithm of the ionic concentration. In this study, the performance of the ion-selective electrode was verified by measuring the EMF of sodium solutions over the concentration range of 10^–4^–10^–2^ M, with standard solutions of sodium chloride.

### Simulation Methods

We have considered all-atomic molecular dynamics simulations of different systems containing pNIPAM chains, Na^+^ and Ph_4_B^–^ ions, and water. The simulations were performed using NAMD software,^27^ and system preparation and postprocessing were done with scripts running in VMD.^28^ The employed force field was the CHARMM force field,^29,30^ which was successfully used in previous pNIPAM simulations^25,31^ (we considered the TIP3P model of water, as parametrized in CHARMM). This choice of water model seems to be appropriate for our simulation studies to be performed above the transition temperature. However, it should be noted that for simulation studies trying to study the swollen–collapsed transition more advanced water models need to be used.^32^ For Ph_4_B^–^, we employed the same CHARMM parameters as in our previous works.^24,25^

The employed simulation parameters were standard in NAMD. The equations of motion were integrated every 2 fs, and electrostatic interactions updated every 4 fs. All bonds between heavy atoms and hydrogen atoms were maintained rigid. Short-range electrostatic and Lennard-Jones interactions were computed with a cutoff of 1.2 nm (LJ switching distance of 1.0 nm). For long-range electrostatic interactions, the particle mesh Ewald (PME) algorithm was used taking a grid spacing of 1.0 Å. Full periodic boundary conditions in all directions were employed. The temperature was controlled with a Langevin thermostat using a damping coefficient of 1 ps^–1^. In the simulations employing a barostat, we employed the Nosé–Hoover–Langevin piston with an oscillation period of 100 fs and a decay time of 50 fs.

In our study, we have considered two different types of simulations: (a) simulation of the structure of a pNIPAM particle and (b) simulation of the effect of concentration of Ph_4_B^–^Na^+^.

In the first kind of simulations, we investigated the structure of a pNIPAM particle made of 20 pNIPAM chains of 20 monomers each (400 monomers in total) in water (51 972 water molecules) in the presence of 27 Na^+^ and 27 Ph_4_B^–^ ions (about 1.65 × 10^5^ atoms). The amount of water and thus the size of the simulation box was selected taking into account the compromise of avoiding image interactions while keeping computational cost reasonable.

The simulation was performed in the NpT ensemble at 45 °C and 1 atm, corresponding to the experimental conditions of the particles reported in this paper. In order to build the simulated system, we first consider a small system with only two chains of pNIPAM in water in the presence of Na^+^ and Ph_4_B^–^. Once the chains were aggregated, we built larger systems by adding more pNIPAM chains until we obtained an aggregate made of 20 chains. Once this aggregate was built, we generated a production run of 700 ns to collect data. The average size of each side of the cubic simulation box was 11.8 nm.

In the second kind of simulations, we studied the effect of different concentrations of NaPh_4_B with a fixed amount of pNIPAM chains. Due to the high computational cost of the simulation of a pNIPAM particle of nanometric size, we could not afford to repeat the previous simulation of a full particle in the presence of different salt concentrations. Instead, we performed the exploration of the effect of salt, considering a simplified system mimicking a large pNIPAM particle. The system consists of a central slab of a hydrophobic material (the same considered in our previous work in ref (24)) covered with adsorbed pNIPAM chains. The slab has an area of 11.38 nm^2^ and a thickness of 1.2 nm, and it was covered by pNIPAM at both sides (10 pNIPAM chains of 20 monomers each, which extend up to 3 nm of the surface). The pNIPAM chains were in contact with different amounts of water and NaPh_4_B. We considered eight different concentrations, from 4 to 27 Na^+^ and Ph_4_B^–^ ions, and sizes of the water regions ranging from 5.5 to 8 nm. In all cases, the simulation was performed in the NpT ensemble (the hydrostatic pressure *p* was controlled only in the direction perpendicular to the surface) at 45 °C and 1 atm, corresponding to the experimental conditions of the particles reported in this paper.

The adsorption of ions at pNIPAM was identified from the first peak in the radial distribution function between pNIPAM atoms and the ions, as in our previous work.^25^

## Experimental Results

**Figure 1:**
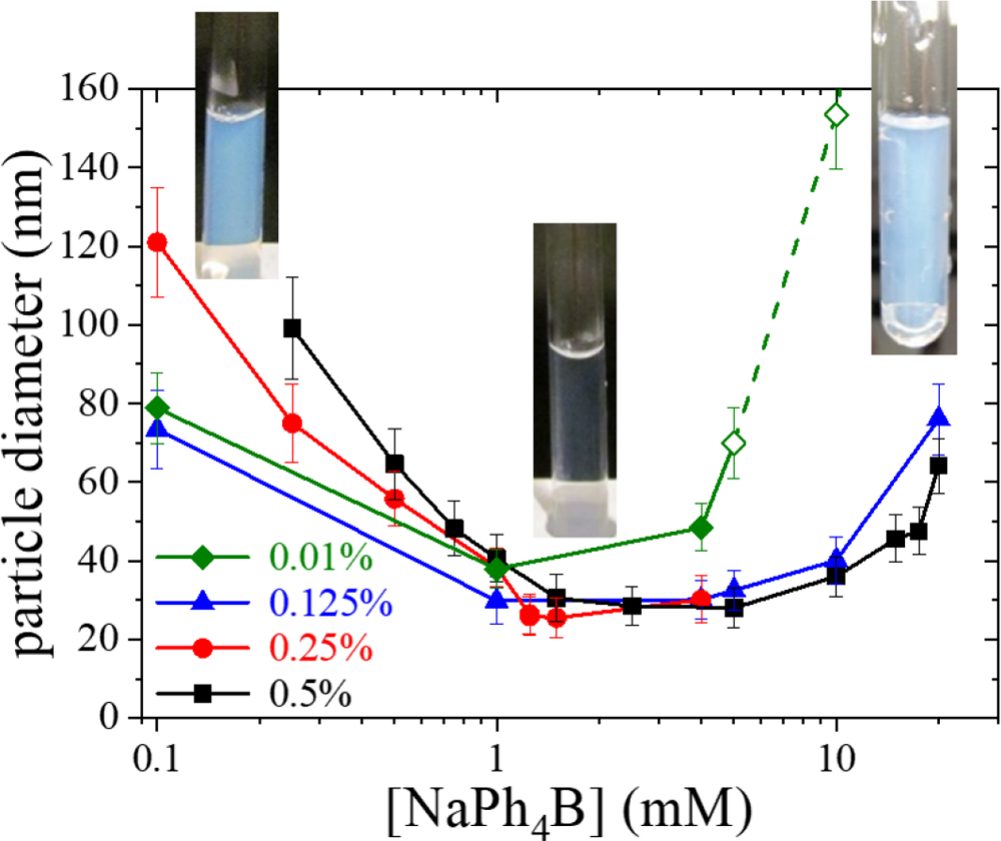
Hydrodynamic diameter of colloidal particles formed upon limited aggregation of pNIPAM in aqueous solutions of different concentrations of NaPh_4_B. *T* = 45 °C. pNIPAM concentration for each data series is indicated in the legend. Open symbols correspond to rapidly evolving (unstable) samples. Insets: Test tube photographs of a few representative samples.

We investigated the state of aggregation of pNIPAM in the presence of NaPh_4_B, by using DLS. Figure 1 shows a set of results obtained for different pNIPAM and NaPh_4_B concentrations, at 45 °C, which is above the LCST of the polymer. As we have pointed out before,^21^ the presence of the hydrophobic tetraphenyl anion has important consequences on the behavior of pNIPAM at high temperatures, limiting its macroscopic phase separation and promoting the formation of submicrometric particles with a well-defined characteristic size. Several features are noteworthy. First, it can be observed that the aggregation is determined by the concentration of both the salt and the polymer. In general, we found that a minimum size is obtained at a specific salt concentration, which depends on the pNIPAM concentration: increasing the pNIPAM concentration shifts the location of this minimum to larger NaPh_4_B concentrations. Second, we found that at lower salt concentrations (below the concentration corresponding to the minimum size) the observed aggregate size increases with pNIPAM concentration. On the contrary, the aggregate diameter was rather independent of pNIPAM concentration for salt concentrations above this minimum and for pNIPAM concentrations above 0.1% w/w. Interestingly, in this regime of relatively large NaPh_4_B concentrations, the system becomes unstable for lower pNIPAM concentrations (<0.1% w/w), when macroscopic phase separation is observed. This significant result is rather counterintuitive: *decreasing the concentration of the dispersed component triggers the instability of the dispersion*. Another feature that must be stressed is that the pNIPAM aggregates are electrostatically stabilized: as we reported before, the addition of an indifferent salt (e.g., NaCl) at enough concentration quickly destabilizes the polymer particles, triggering macroscopic phase separation.

**Figure 2:**
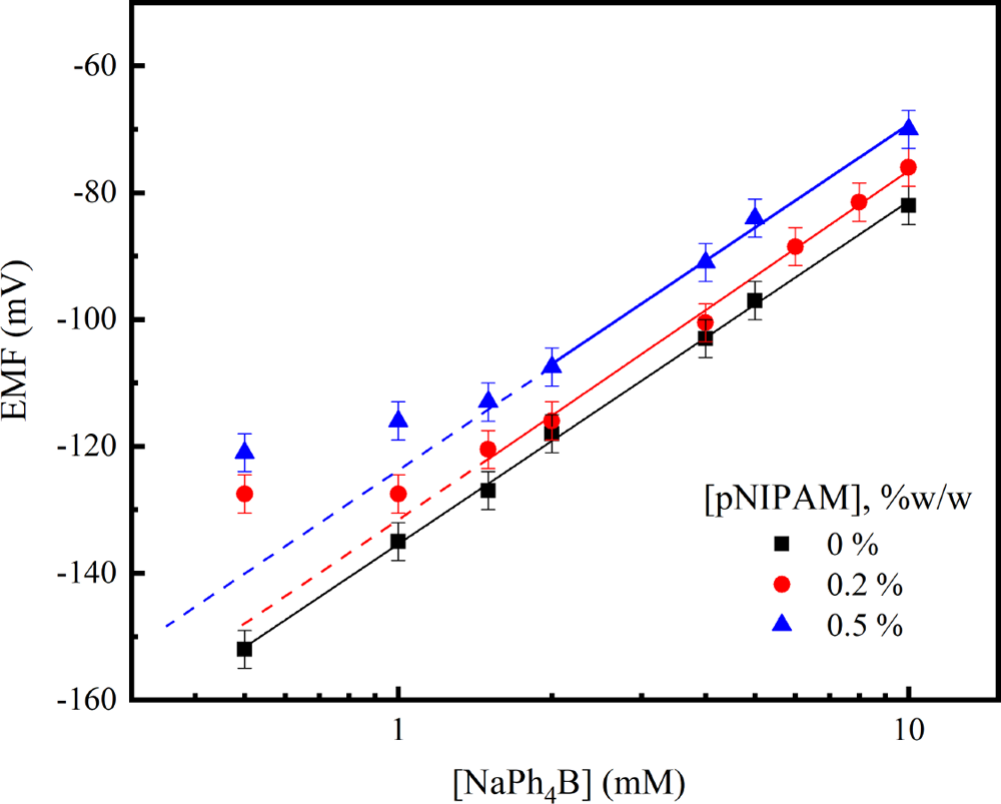
EMF measured with a sodium ISE in aqueous solutions of different concentrations of NaPh_4_B, in the presence of different concentrations of pNIPAM, as indicated. *T* = 25 °C. Continuous lines show the best fit of the data measured at high salt concentrations to Nernst’s equation. The extrapolation of the fit is represented by dashed lines.

The results presented in Figure 1 contain and extend the results previously reported by us in previous studies.^21^ It is important to emphasize that no similar effects are observed in the presence of noninteracting salts (e.g., NaCl). In this case, macroscopic phase separation is always observed at temperatures above the LCST. The observed limited aggregation clearly shows the striking effect of the addition of a hydrophobic anion on the phase behavior of the pNIPAM, indicating a significant interaction of the tetraphenyl anions with the pNIPAM chains. To further characterize this interaction, we have used a sodium-selective electrode described in this section. Figure 2 shows the EMF measured with the sodium ISE for different concentrations of pNIPAM and NaPh_4_B. In the absence of pNIPAM, the measured potential is proportional to the logarithm of the Na^+^ concentration within the range of NaPh_4_B investigated, with a slope *S* of 54 mV, in reasonable agreement with the prediction of Nernst’s equation. Interestingly, the addition of pNIPAM reduces the range of validity of Nernst’s equation: when this polymer is present in the solution, the measured EMF is rather insensitive to changes in Na^+^ concentration at low salt concentrations. This deviation of the Nernstian behavior seems to depend on pNIPAM concentration. For a pNIPAM concentration of 0.2% w/w (0.5% w/w), the measured EMF increases proportionally to the sodium concentrations for NaPh_4_B concentrations above 1 mM (1.5 mM). In addition, in the presence of pNIPAM the measured EMFs were always larger than the values measured in absence of the polymer. It is important to point out that no significant effect of the addition of pNIPAM was observed when solutions of NaCl were investigated for pNIPAM concentrations as high as 0.5% w/w, as reported in the Supporting Information (Figure S1). As the response of the sodium-selective electrode is well described by Nernst’s equation in the presence of NaPh_4_B or a mixture of pNIPAM and NaCl, it can be concluded that neither the pNIPAM nor the tetraphenylborate ion disturbs the performance of the ISE. It is the combination of both species (NaPh_4_B and pNIPAM) that affects the Nernstian response of the electrode at low salt concentrations.

As mentioned before, the measured EMF is a function of the activity of the sodium ions. This activity is the result of the interaction of these ions with all the other components in the solution and, in particular, with the tetraphenyl counterions. However, the distribution of tetraphenyl ions in the solution is mediated by the hydrophobic interaction with the pNIPAM and the entropic force driving a uniformly mixed solution. Thus, it can be argued that the interaction between the tetraphenyl counterions and the pNIPAM chains affects the chemical potential of the sodium ions, as observed by ISE. As the number of Ph_4_B^–^ counterions interacting with pNIPAM is limited by the number of available polymer chains, this effect saturates at a sufficiently large concentration of salt. The ISE results clearly evidence that there exists significant association of the pNIPAM chains with Ph_4_B^–^ anions, even at temperatures well below the LCST.

## Molecular Dynamics Simulations

### Atomistic Description of a pNIPAM Self-Assembled Particle

In order to develop an atomistic description of the pNIPAM particles observed experimentally, we have considered the simulation of a pNIPAM particle with a size of several nanometers, much larger than those considered in previous simulations.^25^ The particle was built by the self-assembly of 20 pNIPAM chains (400 monomers) in water in the presence of 27 Na^+^ and 27 Ph_4_B^–^ ions in water, as described in the Methods section. The statistics were collected over a 700 ns production run.

**Figure 3:**
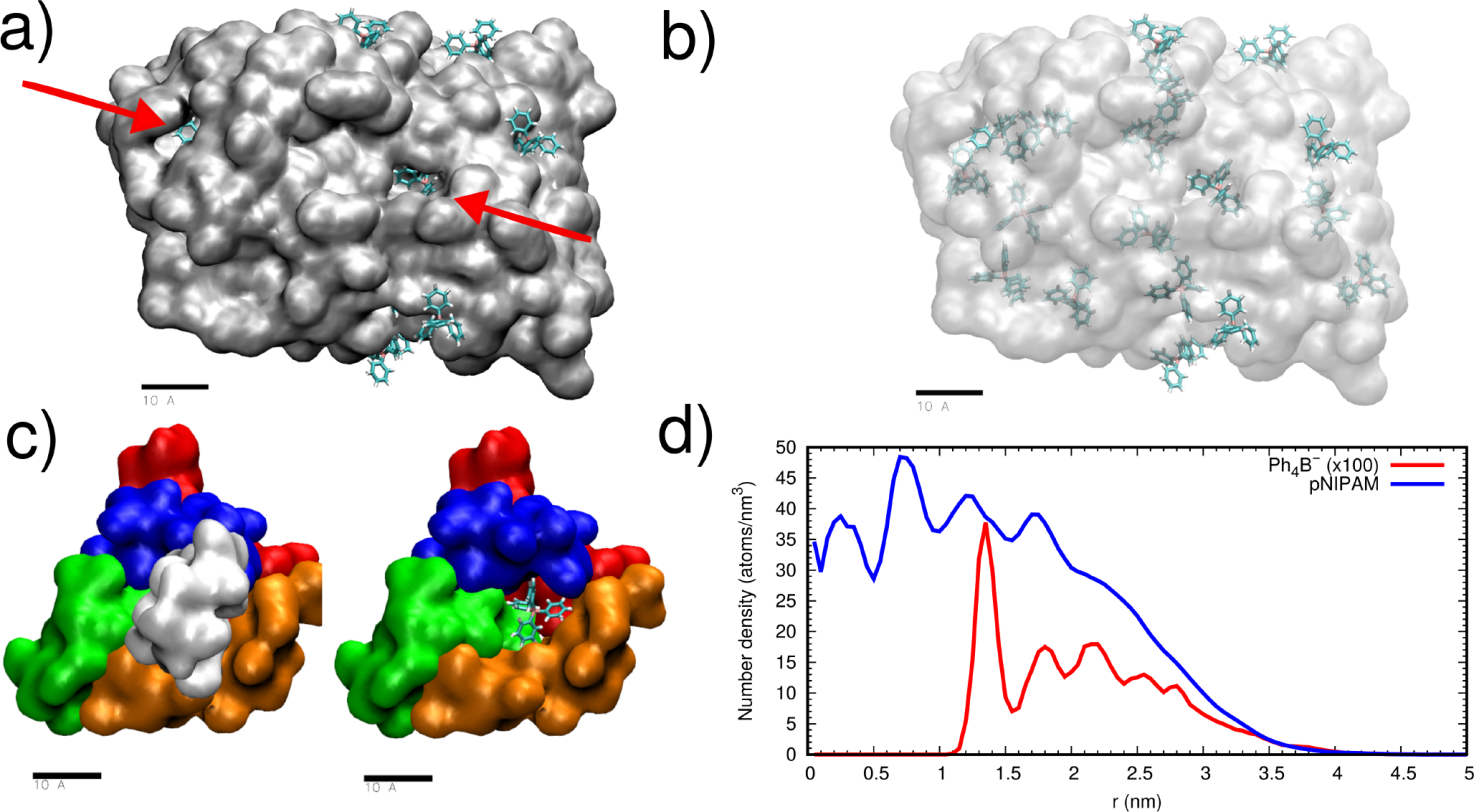
Results from a simulation of a pNIPAM self-assembled particle made of 20 chains (400 monomers) in the presence of Ph_4_B^–^. In all the snapshots, the scale bar corresponds to 1 nm. (a) Snapshot of the pNIPAM particle (shown in surface representation in gray) and adsorbed anions (shown in bonds representation). Water molecules and nonadsorbed ions are not shown for easier visualization. The location of some anions difficult to see is indicated by arrows. (b) Same as (a) but with pNIPAM shown translucid to facilitate the observation of anions located inside the particle (we use depth cueing to better visualize distance to the observer). (c) Zoom-in of the environment of a particular adsorbed anion. Left: all pNIPAM chains (six) in contact with the anion are shown. Right: one of the chains is removed to allow visualization of the internal cavity of pNIPAM containing the anion. We show only the pNIPAM chains in contact with the anion, each one shown in a different color. (d) Internal structure of the pNIPAM particle characterized by the radial density profile (atoms/nm^3^) of non-hydrogen atoms from pNIPAM (blue line) and B atoms from adsorbed Ph_4_B^–^ anions (red line).

As can be seen in the snapshots and the density profile shown in Figure 3, the pNIPAM chains form a self-assembled particle together with adsorbed Ph_4_B^–^ anions. In fact, Ph_4_B^–^ anions not only are adsorbed over the pNIPAM particle but can also be found *inside the particle*, accommodated in nanometric-sized cavities as shown in Figure 3a–c. In this simulation, the average number of Ph_4_B^–^ anions associated with the pNIPAM particle is 17.38 ± 0.02. We have also obtained a very small adsorption of Na^+^, with an average of 0.21 ± 0.01 adsorbed cations. Hence, the particle has acquired a substantial negative charge (∼−17*e*), a consequence of the large affinity of Ph_4_B^–^ for pNIPAM.

The equilibrium density profiles of the pNIPAM chains and Ph_4_B^–^ anions inside the self-assembled particle are shown in Figure 3d (depicted as blue and red lines, respectively). As observed, the particle has a radius of about 4 nm. In addition, the pNIPAM concentration is more or less uniform in the center of the particle, fluctuating around ∼37 atoms/nm^3^, and decays progressively to zero at the interface of the pNIPAM aggregate, with an interface width of about 2 nm. These values for the interface thickness agree with the experimental observations for the interface thickness of collapsed pNIPAM microgels (above the LCST).^33^ The anions can be found inside the particle with a peak of 0.4 ions/nm^3^ at *r* = 1.2 nm and an approximately uniform density of 0.15 anions/nm^3^ for *r* between 1.5 and 3 nm.

The available surface area of the particle (SASA), averaged over the production run, is 250.2 ± 0.1 nm^2^, which corresponds to an average diameter that can be estimated as  nm, consistent with the size estimate obtained from the density profile. As seen in the snapshots of Figure 3, the anions are “engulfed” inside the particle, located in cavities of nanometric size. In order to characterize these internal voids, we have employed a Monte Carlo algorithm designed to identify cavities in biomolecules (such as proteins), implemented in the McVol code.^34^ The algorithm identifies the presence of 18 cavities inside the particle (some of them occupied by anions and others by water), corresponding to a volume of 1.7 nm^3^ out of the 105 nm^3^ total volume of the particle (hence cavities correspond to 1.6% of the particle volume).

Although pNIPAM clusters with the size obtained in the experiments cannot be reproduced in our atomistic MD simulations (because their large size is prohibitive in terms of computation time), our present results, obtained for a cluster of a nanometric size, clearly show that these pNIPAM aggregates acquire an effective charge due to the adsorption of Ph_4_B^–^ anions, which are located not only at the surface of the clusters but also distributed inside them.

### Effect of Concentration

**Figure 4:**
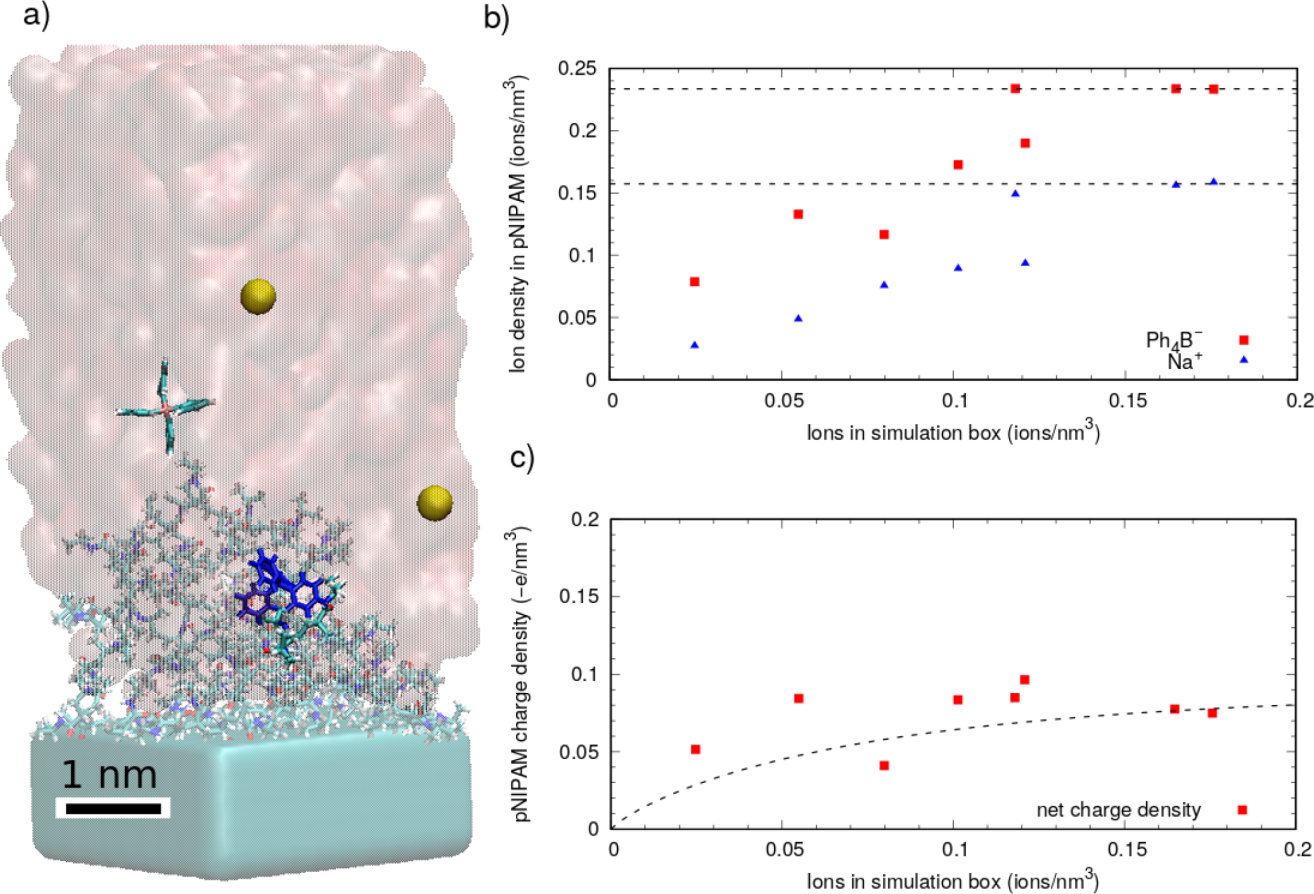
Results of atomistic simulations corresponding to different Ph_4_B^–^Na^+^ concentrations in contact with pNIPAM. (a) Simulation snapshot. (b) Plot of the ion density of anions (*N*_–_^in^/*V*^in^) and cations (*N*_+_^in^/*V*^in^) inside the pNIPAM phase as a function of the total number of ions (anions or cations) in the simulation volume *N*/*V*. The saturation values for each ion are indicated with dashed lines. (c) Plot of the net charge inside pNIPAM as a function of the total number of ions (anions or cations) in the simulation volume *N*/*V*. The dashed line is calculated from the adjustment of the mesoscopic model to the experimental data, as described in the discussion.

As can be observed in Figure 1, the concentration of NaPh_4_B salt plays a key role in the self-assembly of pNIPAM. Due to its computational cost, it is not possible to repeat the simulation described in the previous section for different values of NaPh_4_B concentration. However, it is still possible to use MD simulations to investigate the effect of NaPh_4_B concentration by designing appropriate simulations in a simplified model system. In order to mimic a large pNIPAM particle, we arranged another simulation system consisting of a pNIPAM phase made by 10 pNIPAM chains grafted onto a planar surface and a water phase, at which we initially place the ions (see Figure 4a).

In each simulation, equal numbers *N* of Ph_4_B^–^ and Na^+^ ions were initially placed in the water phase, and the number of ions at each phase (water or pNIPAM) was monitored as a function of time until reaching their final equilibrium value, which typically required about 100 ns of simulation time. We considered eight different simulations, with different ionic concentrations. Once the equilibrium is reached, we computed the average number of anions and cations associated with pNIPAM (denoted by *N*_–_^in^ and *N*_+_^in^, respectively) and the free anions and cations present in the water phase (*N*_–_^bulk^ and *N*_+_^bulk^, respectively) averaged over equilibrium configurations (note that *N*_*i*_^in^ + *N*_*i*_^bulk^ = *N*). We have also computed the average volume *V*^in^ and *V*^bulk^ occupied by the pNIPAM and water phases (*V*^in^ + *V*^bulk^ = *V*), so we can determine the average ion densities at each phase. The charge density acquired by the pNIPAM phase due to the adsorption of ions is also computed as . The results are given in Figure 4b and c.

Our results show that the concentration of Ph_4_B^–^ at the pNIPAM phase (*N*_–_^in^/*V*^in^) is always much larger than the concentration of Na^+^ at the pNIPAM phase (*N*_+_^in^/*V*^in^) (cf. Figure 4b) so the pNIPAM phase always acquires a substantial negative charge density (cf. Figure 4c) that increases as the amount of NaPh_4_B in the simulation box increases. As the number of ions in the simulation increases, their number in the pNIPAM phase also increases until they reach their corresponding saturation values. These saturation values are estimated in Figure 4b, giving
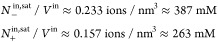
1From these values, we can estimate a saturation value for the charge density of ρ_net_^′sat^ ≈ −0.076 e/nm^3^ = 124 mM. Using these results, we can estimate that a pNIPAM particle with a diameter of say 30 nm (cf. Figure 1) will have at most ∼3300 Ph_4_B^–^ anions and ∼2200 Na^+^ cations associated, with a net negative charge of ∼−1100*e*.

Two significant effects arise from the adsorption of ions by pNIPAM. First, a net electric charge is associated with the pNIPAM chains and aggregates, providing electrostatic stabilization. Second, the effective ionic concentration in the water medium becomes depleted, effectively increasing the effective screening length. Also, the saturation of ion adsorption obtained from simulations has important consequences. The DLS measurements (Figure 1) show that there is a NaPh_4_B concentration at which the mean size of the pNIPAM self-assembled structures (formed at *T* = 45 °C) reaches its minimum value. This situation is related to the saturation concentration, representing the maximum ionic density that the pNIPAM clusters can incorporate. Beyond this salt concentration, no further ions can be associated with the polymer clusters, and the electrostatic stabilization of the particles is compromised. The excess of ions that cannot be adsorbed in the pNIPAM phase contribute to the screening of the electrostatic interaction (instead of increasing electrostatic repulsion). This interpretation is also in agreement with the ISE observations (cf. Figure 2). We will provide a quantitative formulation of this analysis in the next subsection.

### Mesoscopic Theory

In this section, we develop a mesoscopic theory to estimate the net charge (*Q*) of the pNIPAM aggregates arising from the association of Ph_4_B^–^ and Na^+^ ions. This quantity is essential to understand the dependence of the size of the stable pNIPAM aggregates in terms of the salt concentration in the solution, ρ_s_. For this purpose, we will use as input data the results obtained for ρ_net_^′sat^ obtained in the previous section by means of atomistic MD simulations.

The system under study is a solution of pNIPAM chains immersed in water at *T* = 45 °C, with a certain amount of added NaPh_4_B salt. As described before, Ph_4_B^–^ ions show a significant affinity to the pNIPAM chains, driven by the hydrophobic interaction. In addition, as the temperature is higher than the LCST, pNIPAM chains tend to collapse and coagulate.

In our theoretical model, water is treated as a uniform background of relative dielectric constant ϵ = 71.51. The Bjerrum length at this temperature is *l*_B_ = *e*^2^/(4πϵ*k*_B_*T*) = 0.735 nm (*e* is the electron charge, *k*_B_ is the Boltzmann constant, and *T* is the absolute temperature).

The experimental observations indicate the formation of stable clusters of pNIPAM with a well-defined size. The mean radius of these aggregates, *R*, decreases with ρ_s_ for small NaPh_4_B concentration, whereas it increases again for sufficiently large values of ρ_s_, leading to a minimum cluster size for some intermediate salt concentration. This effect has been only observed in the presence of ions with large hydrophobicity such as Ph_4_B^–^, due to the enhanced affinity between these ions and the pNIPAM chains.^21^

To predict the charge of the aggregates and the Debye screening length that governs the extent of the electrostatic interaction, we need to estimate the amount of Ph_4_B^–^ and Na^+^ ions associated with the pNIPAM aggregates. Following the notation used in the previous section, we denote *N*_–_^in^ and *N*_–_^bulk^ as the total number of Ph_4_B^–^ associated with the pNIPAM aggregates and suspended in the bulk solution, respectively. Analogously, *N*_+_^in^ and *N*_+_^bulk^ represent the same quantities, but referred to Na^+^ ions. The corresponding concentrations are given by ρ_*i*_^in^ = *N*_*i*_^in^/*V* and ρ_*i*_^bulk^ = *N*_*i*_^bulk^/*V*, where *V* is the total volume of the system, and *i* = +, −. These concentrations are related by
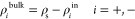
2Here, it should be emphasized that these concentrations are different from the ones obtained in the computer simulations on partitioning, defined as  and  (*i* = +, −), where *V*^in^ and *V*^bulk^ represent the volume of the pNIPAM aggregates and the volume of the bulk, respectively (*V*^in^ + *V*^bulk^ = *V*). If the volume fraction of the pNIPAM phase is denoted by ϕ = *V*^in^/*V*, then the relation between both number densities is
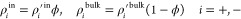
3

In order to provide a theoretical explanation for the nonmonotonic behavior of the cluster size with the NaPh_4_B concentration, we first need to have an estimate of the saturation concentrations consistent with the experimental curve. We denote ρ_–_^sat^ and ρ_+_^sat^ as the saturation concentration of both kinds of ions, defined as the maximum values of ρ_–_^in^ and ρ_+_^in^ that can be reached in the pNIPAM aggregates. These saturation values are defined as the concentration values associated with the pNIPAM aggregates. Thus, the salt concentration outside the aggregates can be adjusted to be much larger than these saturation values.

In addition to these saturation values, we also need to characterize whether the saturation limit is achieved smoothly or sharply. At this point, we need some analytical formula to express ρ_–_^in^ and ρ_+_^in^ in terms of the added salt concentration, ρ_s_. Here, we propose the following ansatz:
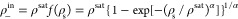
4
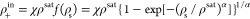
5where ρ^sat^ is the saturation salt concentration and χ < 1. The above-defined function *f*(ρ_s_) depends on both parameters ρ^sat^ and α.

**Figure 5:**
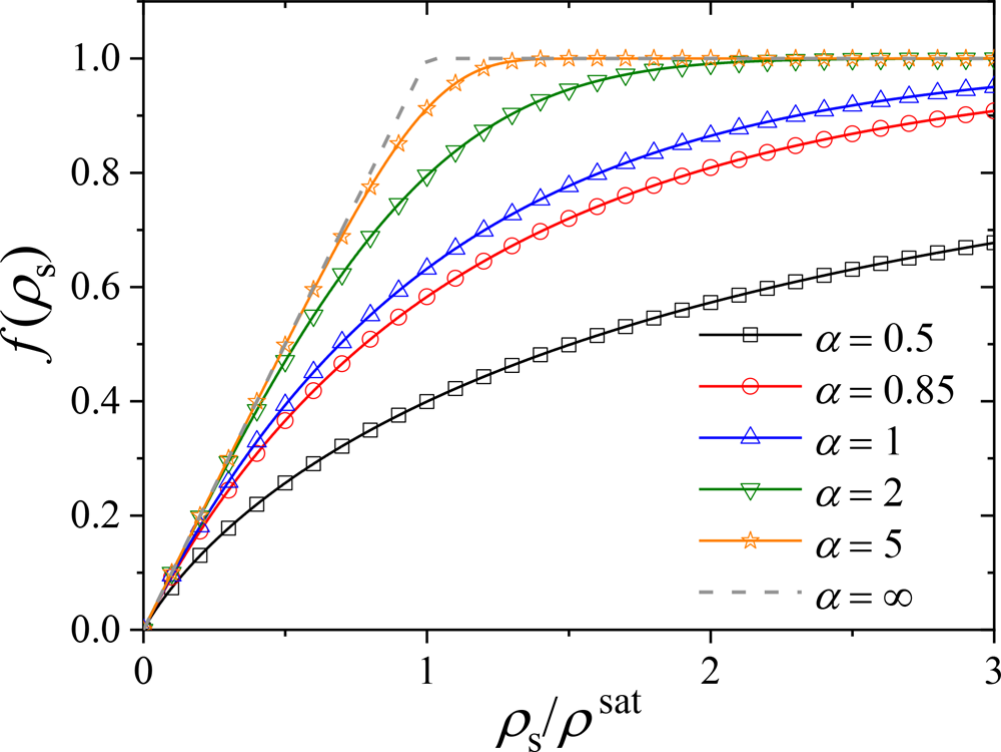
*f*(ρ_s_) as a function of ρ_s_/ρ^sat^. Parameter α controls the steepness at which the saturation concentration, ρ^sat^, is achieved.

Figure 5 depicts *f*(ρ_s_) for different values of the exponent α. As observed, it gathers the most important properties required to describe the association of ions with the aggregates observed in the MD simulations. On the one hand, it provides the right trends in the limit of low and large salt concentrations. Indeed, for ρ_s_ ≪ ρ^sat^, we find *f*(ρ_s_) ∼ ρ_s_/ρ^sat^, which leads to ρ_–_^in^ ∼ ρ_s_ and ρ_+_^in^ ∼ χρ_s_. In other words, for small amounts of added salt all the highly hydrophobic Ph_4_B^–^ ions become incorporated into the pNIPAM clusters by virtue of their strong preference to the pNIPAM phase, whereas only a fraction of Na^+^ in solution (given by χ) migrates nearby or inside the pNIPAM phase because these ions do not interact hydrophobically with pNIPAM. On the other hand, for ρ_s_ ≫ ρ^sat^, *f*(ρ_*s*_) → 1, the pNIPAM clusters reach charge saturation, i.e., ρ_–_^in^ → ρ^sat^ and ρ_+_^in^ → χρ^sat^. Equations 4 and 5 also satisfy the observations reported by the MD simulation, which indicate that the saturation threshold is achieved at roughly the same salt concentration for both kinds of ions (cf. Figure 4(b)). Parameter α > 0 controls the steepness of the saturation process. For α → *∞* (dashed gray line in Figure 5) the process is abrupt and consistent with the Manning condensation prescription,^35^ whereas decreasing α yields smoother curves.

With this definition, the net charge of a spherical cluster of radius *R* is given by
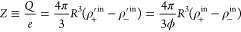
6

**Figure 6:**
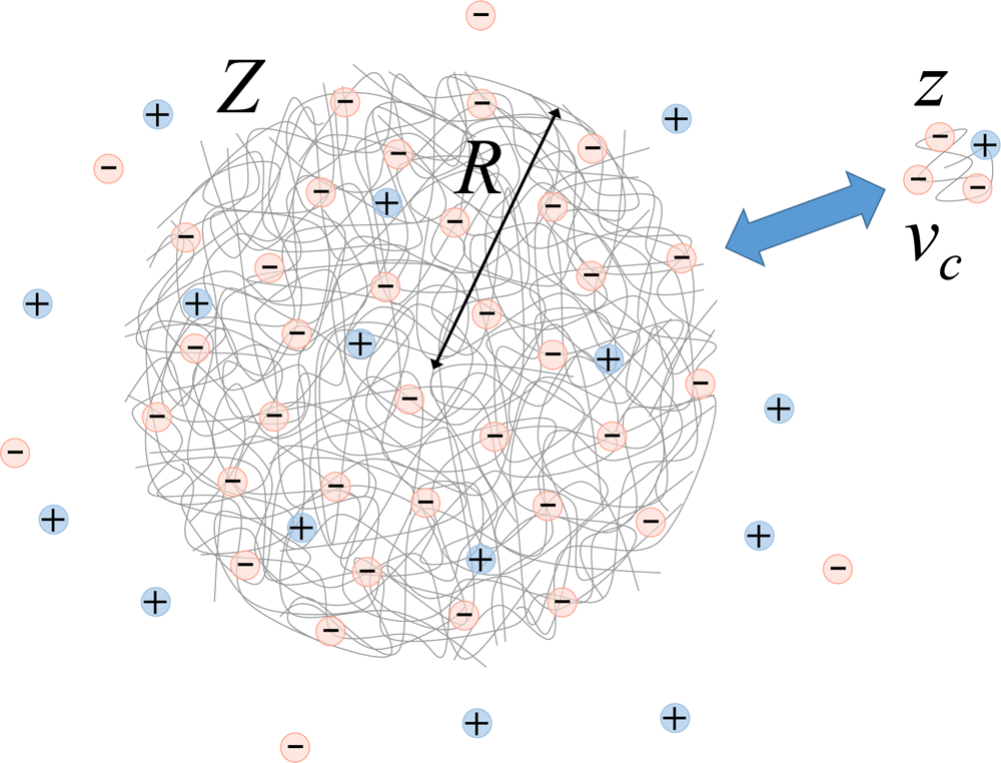
Schematic view of the interaction between a pNIPAM aggregate and a single collapsed pNIPAM chain. These clusters become charge-stabilized due to the adsorption of hydrophobic Ph_4_B^–^ anions.

Similarly, the charge of a single pNIPAM chain is
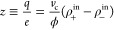
7where *v*_c_ is the volume of a collapsed globular chain. Figure 6 schematically illustrates a pNIPAM aggregate of charge *Q* = *Ze* and radius *R*, interacting with a pNIPAM chain with charge *q* = *ze* and volume *v*_c_. The stability of the aggregates can be analyzed in terms of this interaction, which can be split into two leading contributions. First, the chain experiences a combination of van der Waals and short-range hydrophobic attraction to the aggregate at temperatures above the LCST. For this kind of short-range attraction, the stability of the system does not depend on the specific details of the interaction, so we can safely approximate it by a square-well potential, given by
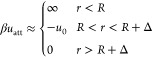
8where β = 1/(*k*_B_*T*). *u*_0_ represents the effective interaction free energy of the chain with the aggregate, involving van der Waals and hydrophobic attractions (which could be on the order of several *k*_B_*T*), and Δ is the typical range of this interaction (on the order of 1 nm).

The second contribution comes from the electrostatic repulsion. Since Ph_4_B^–^ ions are also hydrophobically attracted to the pNIPAM chains, they become associated with the clusters. As shown in the MD atomistic simulations, the adsorption of Ph_4_B^–^ also entails the loading of a certain amount of Na^+^ ions due to the electrostatic attraction. Therefore, this effect yields charged pNIPAM aggregates and charged chains, with net charges given by eqs 6 and 7, which are electrostatically repelled. Assuming a simple Yukawa interaction, we have
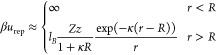
9where κ is the inverse of the Debye screening length. It is important to emphasize that κ, the reciprocal effective electrostatic screening length, only depends on the concentration of ions in the bulk solution (nonassociated with pNIPAM), as
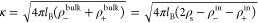
10Using eqs 4 and 5, κ can be written as
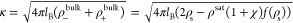
11

When the aggregate size *R* is large enough so that the hydrophobic and van der Waals attraction strength, −*u*_0_, is balanced by the repulsive electrostatic barrier at *r* = *R*, further incorporation of pNIPAM chains to the aggregate is severely hindered:
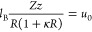
12

This equation represents the stability condition of the pNIPAM aggregates. Inserting eqs 6 and 7, we find that this condition can be rewritten as
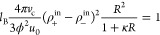
13

Using again eqs 4 and 5, the stability condition reads as
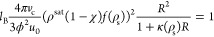
14

In order to remove from this equation the parameters *v*_c_ and *u*_0_, we consider the reference salt concentration ρ_s0_ at which the aggregate radius reaches its minimum value, *R*_0_. It satisfies
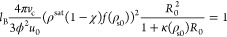
15Dividing eq 14 by eq 15, we find
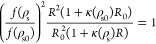
16This equation can be simplified by defining
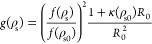
17

Form this, we find a quadradic equation for *R*:
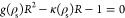
18which finally leads to the following analytical expression for the size of the pNIPAM aggregates as a function of the salt concentration:
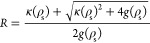
19

It may be shown that eq 19 predicts the existence of a minimum, consistent with the experimental observation. However, in order to compare the predictions of eq 19 with the experimental results, we need to extract from the experimental data the location of the minimum, that is, ρ_s0_ and *R*_0_, as discussed in the following section.

## Discussion

The experiments on formation of pNIPAM aggregates for different salt concentrations were performed in an aqueous suspension at *T* = 45 °C. We explore three samples with different values of pNIPAM mass fractions, *m*_f_ = 0.125% w/w, 0.25% w/w, and 0.5% w/w. Using the density of water and pNIPAM at this temperature ( g/cm^3^ and ρ_m_^NIPAM^ = 1.1 g/cm^3^, respectively), and the fact that, in the collapsed state, pNIPAM aggregates have an internal polymer volume fraction of about ϕ_p_ = 0.6,^36^ ϕ can be obtained from
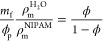
20This leads to ϕ = 1.9 × 10^–3^, 3.74 × 10^–3^, and 7.46 × 10^–3^, for the three mass fractions mentioned above.

**Figure 7:**
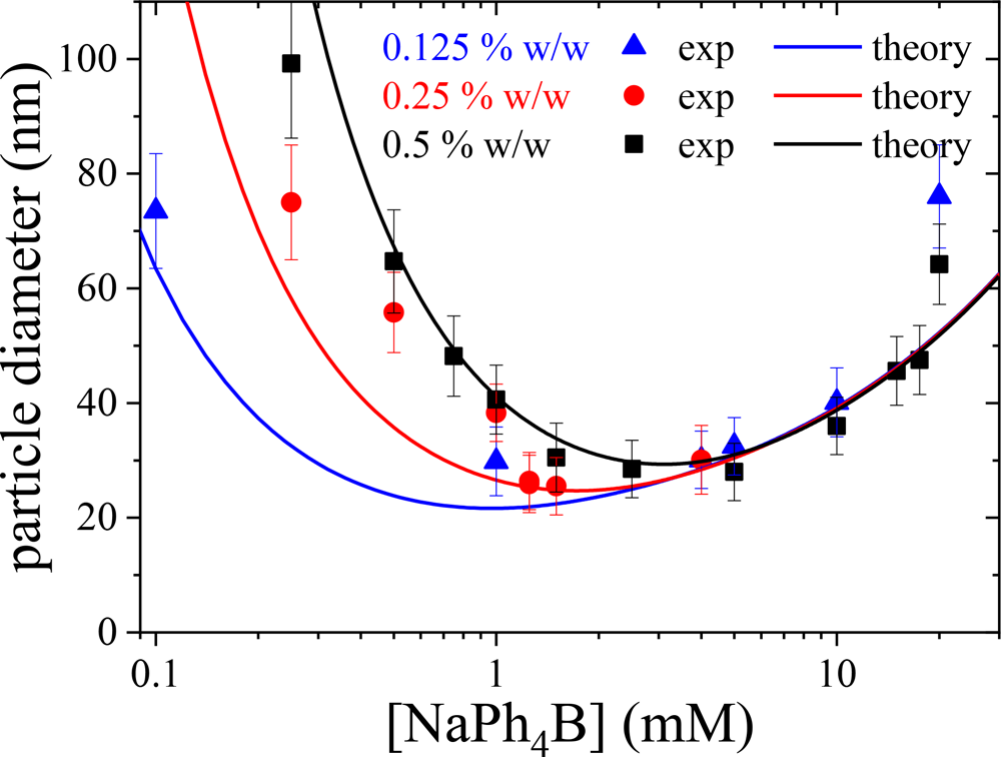
Symbols: mean diameter of the stable pNIPAM aggregates obtained via DLS measurements at *T* = 45 °C, as a function of the NaPh_4_B concentration for three different mass fractions, *m*_f_ = 0.125% w/w, 0.25% w/w, and 0.5% w/w. Solid lines: theoretical predictions for the pNIPAM size obtained from the mesoscopic theory (eq 19).

Blue triangles, red circles, and black squares depicted in Figure 7 show the mean diameter of the pNIPAM aggregates obtained in the DLS experiments, 2*R*, as a function of the salt concentration, ρ_s_. As observed, in all cases, the aggregate size shows a minimum located at some intermediate salt concentration. Interestingly, increasing the pNIPAM mass fraction shifts the position of the minimum to larger NaPh_4_B concentrations. A qualitative explanation of this behavior can be advanced in terms of the interplay between the particle-chain hydrophobic and van der Waals attractions and the electrostatic repulsion, as discussed before. In the absence of NaPh_4_B the attraction between the pNIPAM chains leads to the complete aggregation of the pNIPAM chains. For small salt concentrations, Ph_4_B^–^ anions are strongly attracted to the pNIPAM chains. When the pNIPAM chains aggregate, negatively charged clusters are formed, due to the excess of Ph_4_B^–^ anions (compared to the Na^+^ cations) associated with the pNIPAM cluster. Therefore, increasing the aggregate size also raises its electric charge, *Q*. When *Q* is large enough (at a well-defined mean radius, *R*), the aggregate–chain electrostatic repulsive barrier surpasses the hydrophobic attraction, leading to the stabilization of the pNIPAM cluster against the incorporation of additional polymer chains. At salt concentrations below the saturation level, a larger number of Ph_4_B^–^ ions become associated with the polymer chains when ρ_s_ is increased. This endows pNIPAM aggregates with a larger net charge, which reduces the typical cluster size due to electrostatic stabilization. Therefore, the increase of the aggregate net charge appears as the main reason for the decrease of *R* with ρ_s_.

Increasing even more the NaPh_4_B concentration eventually leads to the charge saturation of the pNIPAM chains. If ρ_s_ is raised above the saturation value, the excess of Ph_4_B^–^ and Na^+^ ions remain suspended in the bulk solution, contributing to the screening of the cluster–chain electrostatic repulsion. This screening reduces the chain–particle electrostatic repulsive interaction, so that the size of the pNIPAM aggregates increases with ρ_s_. As a result of the interplay between both competitive mechanisms, the curve *R* = *R*(ρ_s_) develops a local minimum located close to the saturation value.

The fitting of the experimental data using eq 19 is performed as follows: We first consider the DLS data for *m*_f_ = 0.5% w/w (ϕ_0_ = 7.46 × 10^–3^), shown as black squares in Figure 7, for which the value of the cluster radius at the minimum is *R*_0_ = 15.5 nm. Then, we use ρ^sat^ and the exponent α as the only two fitting parameters. χ does not represent an additional independent fitting parameter, as it is deduced from the MD simulations. Indeed,  mM = ρ^sat^(1 – χ)/ϕ, allowing the calculation of χ from ρ^sat^. The best fitting of this set of experimental results is achieved for ρ^sat^ = 1 mM and α = 0.85. This calculated saturation value appears to be in good agreement with the ISE results. It leads to χ = 0.075, which means that not only hydrophobic Ph_4_B^–^ ions become associated with the pNIPAM aggregates but also a small fraction of Na^+^ tends to emigrate driven by the electrostatic attraction with the negatively charged aggregates. The theoretical prediction, shown as a solid black line in Figure 7, is in good quantitative agreement with the DLS measurements. In particular, the theory predicts the existence of a minimum size of the pNIPAM aggregates, located at ρ_s_ = 3.1 mM, which is consistent with the experimental data within the experimental accuracy.

The exponent α controls the steepness of the transition toward ion saturation inside the pNIPAM phase. The value α = 0.85 implies that the net charge of the pNIPAM aggregates increases very smoothly with the added salt concentration. This is also consistent with the results obtained with MD simulations, which also predict a very smooth growth of the net charge density inside the pNIPAM phase. In fact, using α = 0.85 leads to the dashed line in Figure 4(c), in good qualitative agreement with the simulation data.

In the second fitting step, we determine the theoretical predictions for two more diluted samples, namely, *m*_f_ = 0.125% w/w and *m*_f_ = 0.25% w/w. In both cases, the values of α = 0.85 and χ = 0.075 are fixed, and the salt concentration at saturation is rescaled by the new pNIPAM volume fraction, i.e., ρ^sat^ → (ϕ/ϕ_0_)ρ^sat^, leading to 0.255 and 0.502 mM, respectively. That is, we do not use additional fitting parameters to obtain these new predictions. The theoretical results obtained using these parameters in eq 19 are shown as blue and red lines in Figure 7. As can be observed in the figure, the agreement between theory and DLS measurements is in general very good. In particular, the theory is able to capture the shift of the minimum in aggregate size toward small salt concentrations upon decreasing pNIPAM concentration. This shift comes from the fact that more dilute pNIPAM suspensions require smaller salt concentrations to reach saturation. The mesoscopic model also predicts a polymer-concentration dependence of the preferred aggregate size at salt concentrations below the saturation value. More significantly, it also predicts a similar behavior of the three samples in the regime of large salt concentrations, which is in good agreement with the experimental observations. This convergence may be explained again in terms of saturation. Once the salt concentration is large enough compared to ρ^sat^, the charge of the pNIPAM aggregates is independent of salt concentration, and the preferred aggregate size is independent of polymer or salt concentration.

In summary, the good agreement between DLS experimental results and our mesoscopic theory confirms that the dependence of the pNIPAM aggregate size on salt concentration is indeed led by the competition between hydrophobic attractive interactions and the electrostatic repulsion induced by the specific association of hydrophobic Ph_4_B^–^ ions. This ion-induced limited pNIPAM aggregation is not observed in the presence of nonhydrophobic (nonassociative) anions. Nevertheless, our simple theoretical model does not consider charge renormalization effects that may lead to a smaller effective charge of the pNIPAM aggregates, especially in the regime of large salt concentrations.^37^ The comparison between theory and DLS experiments indicates that this effect could be responsible for the increase of the particle diameter of the pNIPAM clusters observed in the experiments for NaPh_4_B concentrations above 20 mM, which is not captured by our analytical theoretical model. This reduction of the effective charge is expected to become even more significant for smaller amounts of pNIPAM, such as *m*_f_ = 0.01% w/w. Indeed, in this case, the saturation regime is attained at about ρ^sat^ ∼ 0.1 mM. For large enough salt concentrations, this system experiences an important increase of the ionic concentration in the bulk that reduces the effective charge of the pNIPAM aggregates, inducing the destabilization of the pNIPAM aggregates against coagulation for large salt concentrations, as observed in the data depicted in Figure 1.

The simple mesoscopic model just described explains how limited pNIPAM aggregation, accompanied by the formation of particles of well-defined size, is the consequence of the size-dependent interaction between a single chain and a growing aggregate, which results from the balance between the hydrophobic attraction and the repulsive electrostatic interaction mediated by the effective screening length. However, to assess the stability of the global system, we must also consider the interaction between two aggregates. As a first approximation, we can estimate this interaction by adding the hydrophobic attraction (i.e., as suggested by Tabor and co-workers^38^) to the DLVO interaction energy between two spheres.^39^ Once again, to describe the electrostatic repulsion between charged aggregates, the effective Debye screening length (or effective ionic strength) must be integrated, taking into account the association between the ions and the polymer aggregates. Estimated calculations of the particle–particle interaction, obtained by adding the different contributions just mentioned, are presented in the Supporting Information (Figure S2). It can then be understood why by reducing pNIPAM concentration the system becomes unstable, which at first sight appears counterintuitive. At shorter Debye lengths (due to higher effective salt concentrations), the fine balance between attractive hydrophobic and repulsive electrostatic forces is shifted toward the attractive side, and the particles coalesce. On the contrary, if the effective screening length increases (by increasing the polymer concentration, which translates into a reduction of the effective salt concentration), the electrostatic repulsion will be enhanced, and the coalescence between pNIPAM aggregates will become increasingly difficult.

## Conclusions

In this work, we have explored the implications of the weak anion–pNIPAM association driven by the hydrophobic interaction on the polymer phase behavior. We have shown that polymer chains are associated with hydrophobic ions even at temperatures below the LCST, when the polymer chains are well hydrated. The effect of this association is twofold. First, it provides an effective electric charge to the macromolecular chains. Second, it diminishes the effective salt concentration in the solvent. By these means, at temperatures above the LCST macroscopic phase separation of the polymer is restricted, and submicrometric, polymer-rich particles are formed.

Our results show the existence of a saturation concentration for the association of NaPh_4_B with polymer chains. Below the saturation point, increasing the salt concentration enhances the adsorption of hydrophobic Ph_4_B^–^ ions onto the pNIPAM chains, reducing the size of the microparticle due to the larger charge density. Above the saturation point, increasing the salt concentration enhances the screening of the electrostatic interaction, reducing the repulsion between pNIPAM particles and chains. Consequently, the hydrophobic attraction increasingly dominates over the electrostatic repulsion, leading to the growth of the pNIPAM aggregates. Thus, the competition between the stabilizing electrostatic repulsion and the destabilizing interchain hydrophobic interaction leads to a nonmonotonic behavior of the size of the pNIPAM aggregates with increasing salt concentration.

The specific adsorption of Ph_4_B^–^ onto the pNIPAM phase has been corroborated by atomistic molecular dynamic computer simulations, which clearly show that this hydrophobic ion is strongly attracted to the pNIPAM chains, not only at the surface but also at the interior of the pNIPAM aggregates. These simulations are also able to reproduce the charge saturation effect observed in the experiments. Finally, based on this reentrant stabilization mechanism, a mesoscopic theoretical model has been developed, consistent with the atomistic simulations, which provides good quantitative predictions for the size of the pNIPAM particles for several concentrations of pNIPAM chains. We believe that the process described in this work opens avenues for the dynamic control of the formation and size of monodisperse polymer microparticles and can be extrapolated to understand the limited phase separation of other macromolecular systems in a complex environment.

Conceptually, our work shows that the behavior of polymer solutions (in this case, pNIPAM) can be controlled and tuned by specific interactions with particular types of ions or molecules (in this case, with the highly hydrophobic Ph_4_B^–^). This general concept is not exclusive to this specific system. For example, it is in line with previous results obtained with other polymer systems such as carbohydrate polymer dispersions,^40^ in which the presence of certain solutes (mostly kosmotropic species) modify substantially the hydration of the macromolecules and allow an external control of the interactions between the chains. All these results also emphasize the idea that specific ionic interactions in polymer solutions induce a much richer behavior than that observed in the typical systems considered in the literature of specific ionic effects^23,41^ such as colloids, proteins, or surfaces.

## Supporting Information Available

Additional results obtained with the ion-specific electrode and estimated calculations of the interparticle interaction energy (PDF)
