# Supplementary material for: Interaction-Limited Aggregation: Fine-Tuning the Size of pNIPAM Particles by Association with Hydrophobic Ions
Source: Macromolecules. 2023 Mar 17;56(6):2246–57. doi: 10.1021/acs.macromol.3c00132 (PMC10064791; doi:10.1021/acs.macromol.3c00132)
Supplement: Supplementary file 1 — ma3c00132_si_001.pdf [file ma3c00132_si_001.pdf]

Supporting Information for the article Interaction-Limited Aggregation: Fine-tuning the Size of pNIPAM Particles by Association with Hydrophobic Ions

Jordi Faraudo,[1*] Arturo Moncho-Jordá,[2,3] Delfi Bastos-González,[2] and Carlos Drummond[4*]

1 Institut de Ciència de Materials de Barcelona (ICMAB-CSIC), Campus de la UAB, E-08173 Bellaterra, Spain

2 Biocolloid and Fluid Physics Group, Department of Applied Physics, University of Granada, Av. Fuentenueva 2, E-18001 Granada, Spain

3 Institute Carlos I for Theoretical and Computational Physics, Facultad de Ciencias, Universidad de Granada, Campus Fuentenueva S/N, 18071 Granada, Spain

4 Centre de Recherche Paul Pascal Univ. Bordeaux, CNRS, CRPP, UMR 5031, F-33600 Pessac, France

Additional results obtained with the Ion Specific Electrode

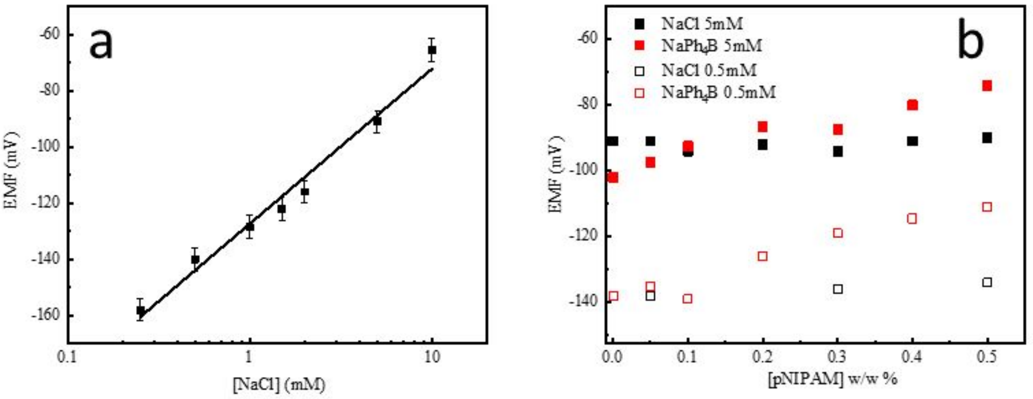

Figure S1. a) EMF measured with a sodium ISE in aqueous solutions of a) NaCl. The continuous line shows the best fit of the measured data to Nernst's equation.[1] The slope S of the line is 55.2 mV, in reasonable agreement with the prediction of this equation. b) NaCl and $NaPh_4B$ in presence of different concentrations of pNIPAM, as indicated. T = 25˚C. The measured EMF is independent of the presence of pNIPAM in the case of NaCl. On the contrary, the polymer affects the values measured for $NaPh_4B$, as discussed in the main text.

Calculated interparticle interaction energy

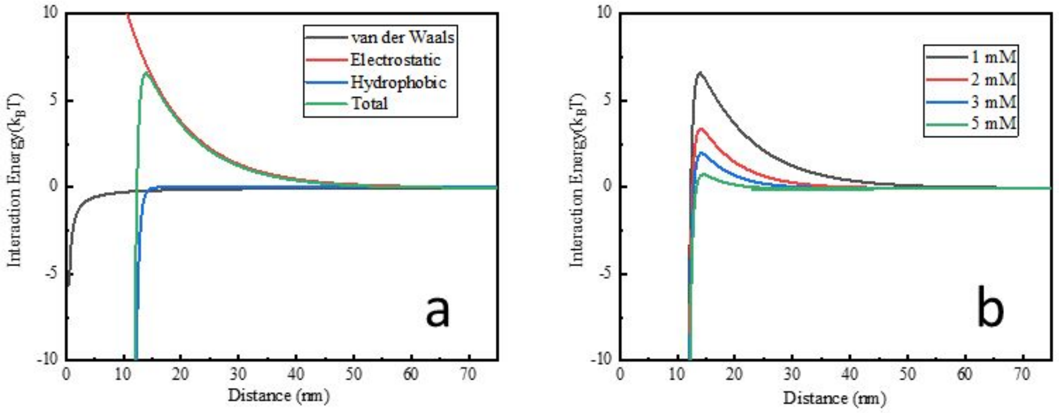

Figure S2. (a) Particle-particle energy of interaction between pNIPAM particles of radius 25 nm in a 1mM salt solution, calculated by adding up the attractive (van der Waals and hydrophobic) and repulsive (electrostatic) contributions. A non-monotonic behavior, with an energy barrier that will slow down interparticle aggregation, is obtained. (b) Total interaction energy for different values of the effective ionic strength. The energy barrier is critically dependent on the effective ionic strength in the media, as discussed in the main text. The larger the energy barrier the more stable the pNIPAM particles will be.

Van der Waals and electrostatic contributions were estimated as described by Israelachvili,[2] considering the electrostatic potential of the particles ψ_0= 35 mV (typical measured ζ-potential value of the pNIPAM aggregates; measured in a Zetasizer, Malvern), and the effective Hamaker constant A=0.55 $\times 10^{-20}$J, calculated from the dielectric constant and refractive index of pNIPAM and water, considering a volume fraction of pNIPAM in the particles ϕ = 0.6.[3] The hydrophobic contribution was assumed to exponentially decay with the inter-particle separation, as proposed by Tabor and coworkers,[4] considering the pNIPAM-water interfacial free energy[5] of 5 mJ/m^2 and a characteristic length for the hydrophobic interaction of 0.5 nm.

References

(1) Bard, A. J.; Faulkner, L. R. *Electrochemical Methods: Fundamentals and Applications. 2nd Edition*, Second.; Sons, J. W. &, Ed.; New York, 2001.

(2) Israelachvili, J. N. J. N. *Intermolecular and Surface Forces*, Third.; Academic Press, 2011.

(3) Kujawa, P.; Aseyev, V.; Tenhu, H.; Winnik, F. M. Temperature-Sensitive Properties of Poly(N-Isopropylacrylamide) Mesoglobules Formed in Dilute Aqueous Solutions Heated above Their Demixing Point. *Macromolecules* **2006**, *39* (22), 7686–7693. https://doi.org/10.1021/ma061604b.

(4) Tabor, R. F.; Wu, C.; Grieser, F.; Dagastine, R. R.; Chan, D. Y. C. Measurement of the Hydrophobic Force in a Soft Matter System. *J. Phys. Chem. Lett.* **2013**, *4* (22), 3872–3877. https://doi.org/10.1021/jz402068k.

(5) Liang, X.; Nakajima, K. Nanofishing of a Single Polymer Chain: Temperature-Induced Coil–Globule Transition of Poly(N-Isopropylacrylamide) Chain in Water. *Macromol. Chem. Phys.* **2018**, *219* (3), 1–6. https://doi.org/10.1002/macp.201700394.
